# Unsupervised Acoustic Anomaly Detection for Rotating Machinery Under Submarine-like Environments: Considering Data Scarcity and Background Noise via Proxy Data Generation

**DOI:** 10.3390/s26092659

**Published:** 2026-04-24

**Authors:** Kwang Sik Kim, Jang Hyun Lee

**Affiliations:** 1 Extreme Technology Research Center for Ship and Offshore Platform, Inha University, Incheon 22212, Republic of Korea; kskim@inha.ac.kr; 2 Department of Naval Architecture and Ocean Engineering, Inha University, Incheon 22212, Republic of Korea

**Keywords:** submarine equipment, unsupervised acoustic anomaly detection, physics-motivated noise modeling, proxy data (MIMII dataset), signal-to-noise ratio (SNR) augmentation, edge computing, Gaussian mixture model (GMM), ensemble autoencoder

## Abstract

This study proposes a noise-robust unsupervised acoustic anomaly detection framework for early identification of abnormal operating conditions in rotating machinery under submarine-like environments with severe data scarcity. In such environments, underwater background noise and onboard interference sources significantly degrade signal quality, while limited computing resources constrain the deployment of high-complexity deep learning models. To address the lack of labeled fault data, the publicly available MIMII dataset was adopted as a proxy platform, and representative submarine interference sources were physically modeled, including colored background noise, structure-borne resonance, band-limited auxiliary noise, tonal components, and sensor noise. These components were combined and scaled to predefined SNR levels (−6 to 6 dB) to generate realistic noise-augmented data. Three unsupervised approaches were compared under edge deployment constraints: (i) Gaussian Mixture Model (GMM) with statistical MFCC features, (ii) statistical-feature-based Ensemble Autoencoder, and (iii) Conv1D-based Ensemble Autoencoder using 1-s log Mel-spectrogram segments. Performance was evaluated in terms of AUC, F1-score, and computational cost. Results show that GMM provides competitive detection performance with minimal computational burden, whereas Conv1D achieves superior accuracy when temporal fault patterns dominate, at the expense of higher complexity. The study provides practical design guidelines for acoustic anomaly detection under multi-noise and resource-constrained conditions.

## 1. Introduction

Submarines are complex weapon systems designed to execute prolonged underwater missions, and the operational integrity of their propulsion and auxiliary equipment is critical to the survivability of the overall platform. Therefore, in submarine operations, condition monitoring procedures that enable early detection of abnormal symptoms during operation are essential to prevent catastrophic failures, rather than relying solely on corrective maintenance after a fault has occurred [1]. Traditionally, maintenance has relied on time-based strategies, which do not adequately reflect real-time degradation or abnormal conditions [2]. As a result, they do not sufficiently account for performance degradation caused by varying operational conditions or reflect real-time abnormal signatures [2]. To address these limitations, Condition-Based Maintenance (CBM) and Prognostics and Health Management (PHM) technologies—which leverage sensor data to monitor equipment conditions and predict potential faults—have attracted increasing attention [3,4,5,6].

In security-sensitive domains such as submarine systems, the acquisition and sharing of labeled fault data are inherently limited due to strict confidentiality constraints. In addition, high system reliability and preventive maintenance policies result in a scarcity of fault occurrences, leading to severe data imbalance. Under these conditions, supervised learning approaches that rely on labeled fault data are difficult to apply, making unsupervised anomaly detection a more practical alternative. Nevertheless, even unsupervised models require representative data for validation, necessitating the construction of proxy datasets that can emulate realistic operating conditions.

Acoustic anomaly detection in submarines faces environmental constraints beyond data scarcity, particularly in terms of noise and limited computing resources. Submarines are continuously subjected to irregular and persistent environmental noise originating from fluid flow, propulsion systems, and onboard machinery. Such environmental noise masks the acoustic signatures of target equipment, reduces the signal-to-noise ratio (SNR), and consequently increases the likelihood of false alarms or missed detections [7,8]. Additionally, due to constraints related to limited onboard space, power availability, and thermal management, submarines cannot rely on high-performance server infrastructures commonly available in terrestrial environments. As a result, real-time processing must be performed on small-scale embedded or edge computing platforms [9]. Although large-scale deep learning models and computationally intensive preprocessing techniques can help mitigate high-noise conditions and domain shift, they are often impractical in resource-constrained edge environments. Therefore, practical deployment requires a lightweight preprocessing pipeline capable of efficiently handling large volumes of acoustic data, along with computationally efficient algorithms suitable for real-time operation.

Considering the practical constraints of data scarcity, complex noise environments, and limited onboard computing resources, this study proposes a lightweight unsupervised acoustic anomaly detection framework based on proxy data to emulate submarine operating conditions. First, a lightweight preprocessing pipeline is designed using a proxy dataset constructed to reflect submarine-like environments. Leveraging the publicly available rotating machinery dataset (MIMII), multiple noise components observed in submarines—including background noise, structure-borne noise, auxiliary machinery noise, tonal components, and sensor noise—are modeled according to their physical origins and synthetically combined to replicate operational acoustic conditions. Next, under diverse noise scenarios and constrained computing capabilities, a noise-robust and computationally efficient preprocessing pipeline is developed to minimize information loss while reducing computational complexity. Finally, representative unsupervised learning algorithms are comparatively analyzed to identify an optimal model that simultaneously satisfies anomaly detection accuracy and computing resource efficiency. Through this procedure, the study highlights the practical feasibility of lightweight acoustic anomaly detection under realistic submarine operational constraints, while providing a systematic framework that integrates proxy data generation, noise modeling, and algorithm selection for resource-constrained environments.

### 1.1. Literature Review

Real-time fault and anomaly monitoring has increasingly evolved toward the analysis of sensor data using machine learning or deep learning models to detect fault-related patterns [3,5,10]. Comprehensive reviews on data-driven machinery health monitoring and prognostics have also highlighted the growing role of machine learning and signal-processing-based approaches in modern PHM systems [10,11]. To ensure diagnostic reliability, Failure Mode and Effects Analysis (FMEA) should first be conducted to identify critical components and select physical signals that most effectively reflect equipment conditions [12,13,14]. While vibration signals are widely used for condition monitoring, acoustic signals have also been shown to contain useful diagnostic information through variations in acoustic radiation patterns. In particular, acoustic sensing provides a practical alternative in environments where direct sensor installation is limited or structural configurations are complex, since acoustic measurements enable non-contact monitoring of equipment conditions [14,15].

With the advancement of deep learning technologies, numerous fault diagnosis models have been developed and reported in the literature [4,5,10]. Recent studies have further investigated machine learning frameworks for prognostics and health management (PHM), highlighting the role of transfer learning and data-driven modeling in fault diagnosis under limited data conditions [16]. However, most of these approaches assume supervised learning environments in which normal and fault data are sufficiently and evenly available. In defense weapon systems such as submarines, acquiring or externally sharing actual fault data is structurally infeasible due to stringent security restrictions. Moreover, rigorous preventive maintenance policies and high-reliability design standards result in extremely limited fault occurrences, thereby leading to severe data imbalance issues [1]. To alleviate data imbalance, data generation techniques based on Generative Adversarial Networks (GANs) have been widely explored [17]. However, GAN-based approaches often require significant computational resources and may suffer from training instability, making them less suitable for embedded onboard systems operating under constrained computing environments. Therefore, instead of employing computationally intensive data generation techniques, this study adopts an unsupervised learning-based anomaly detection approach as a more feasible alternative. By learning only normal operating data to construct a boundary of the normal distribution, the model identifies observations that deviate from this learned region as abnormal conditions [18,19].

Anomaly detection in submarine environments must address the fundamental challenge posed by complex acoustic noise conditions. Submarine acoustic environments typically consist of broadband background noise combined with tonal components generated by onboard rotating machinery, which have been widely studied in recent underwater acoustic noise modeling research [7,20]. Furthermore, variations in operational depth and speed can alter the noise distribution, inducing a domain shift phenomenon that degrades model performance by reducing the SNR [21]. To mitigate these issues, previous studies have applied standalone denoising algorithms as preprocessing steps [22] or proposed domain adaptation techniques to address distribution discrepancies between the training (source domain) and operational (target domain) environments [23]. However, these approaches often assume the availability of target-domain data and may introduce additional model complexity. In addition, condition monitoring approaches based on deviations from physically defined operating conditions have also been investigated. In such approaches, abnormal states are identified through changes in statistical signal characteristics derived from sensor measurements [24]. While this approach primarily focuses on operating-condition-based deviations, it does not explicitly consider the influence of complex environmental noise conditions.

To overcome the security restrictions that prevent the use of actual submarine onboard equipment data, this study employs the MIMII dataset—a standard benchmark for rotating machinery acoustics—as proxy data [25]. In particular, the pump category of the MIMII dataset was selected as the representative rotating machinery component, since pump systems are widely used in marine platforms including submarines and surface vessels. Acoustic signals from submarine onboard equipment are influenced by multiple noise sources that can be classified according to their physical origins, including ambient noise, structure-borne noise, and sensor noise [7,26]. This physically grounded categorization provides a foundation for signal modeling aimed at emulating realistic operational environments.

Submarine onboard systems operate under strict constraints in terms of power supply, physical space, and thermal management. Consequently, diagnostic models must be deployed on embedded or edge computing platforms rather than on high-performance server infrastructures, thereby necessitating inherently lightweight architectures [19,27]. Therefore, this study adopts a design-stage optimization strategy that emphasizes computational efficiency from the early stage of model development.

Recent studies have explored the integration of spectrum-based acoustic features with unsupervised learning algorithms to enable real-time deployment in edge computing environments, rather than relying on computationally intensive deep neural networks [28]. In modeling high-dimensional acoustic feature distributions, statistical approaches such as the Gaussian Mixture Model (GMM) offer flexible probability density estimation with significantly lower computational complexity compared to deep learning architectures [29]. Statistical learning approaches such as Gaussian Mixture Models (GMMs) therefore provide an efficient alternative for anomaly detection in resource-constrained embedded environments. Several studies have also systematically evaluated unsupervised anomaly detection algorithms in real-time operational contexts, explicitly analyzing the trade-off between detection performance and computational efficiency [30]. Collectively, these findings suggest that when processing variable-length time-series acoustic signals, statistical pooling-based approaches—such as extracting mean or maximum values along the temporal axis—can serve as effective alternatives to complex recurrent neural networks (RNNs) or large-scale deep architectures. Statistical pooling enables robust summarization of global signal characteristics with minimal computational overhead, making it particularly suitable for real-time anomaly detection in resource-constrained edge environments [31,32]. To better clarify the research landscape and highlight the research gaps identified in the literature, representative studies related to environmental noise modeling, machine learning-based fault diagnosis, and edge-based monitoring systems are summarized in Table 1. The table presents the techniques used in previous studies, their application systems, and their main advantages and limitations.

### 1.2. Research Object

The present study aims to develop and validate a practically deployable unsupervised learning-based acoustic anomaly detection framework under security and data constraints that limit access to actual acoustic measurements from submarine onboard equipment. To address these limitations, this study proposes a proxy-data-based framework designed to emulate realistic submarine acoustic operating conditions. The main contributions of this study can be summarized as follows:

- Proxy data generation for submarine acoustic environments: A proxy data construction strategy is developed to emulate submarine operational acoustic conditions. The publicly available rotating machinery dataset (MIMII) is adopted as a surrogate platform, while representative noise sources observed in submarine-like environments—including background noise, structure-borne noise, auxiliary machinery noise, tonal components, and sensor noise—are modeled according to their physical origins and synthetically combined to construct noise-augmented datasets under multiple SNR conditions.
- Lightweight noise-aware preprocessing framework: A computationally efficient preprocessing pipeline is designed to operate under diverse noise conditions and limited onboard computing resources. The proposed pipeline aims to minimize information loss while reducing computational complexity, making it suitable for embedded or edge computing environments.
- Comparative evaluation of lightweight anomaly detection models: Representative unsupervised models—including a probabilistic GMM using MFCC-based statistical features, a reconstruction-error-based Ensemble Autoencoder, and a Conv1D-based Ensemble Autoencoder designed to capture temporal local patterns—are systematically compared under identical training conditions.
- Trade-off analysis between detection accuracy and computational efficiency: Detection performance metrics (e.g., AUC and F1-score), together with training and inference time and memory usage, are quantitatively analyzed to evaluate the trade-off between anomaly detection performance and computational efficiency in resource-constrained environments.

Through this framework, the study aims to provide practical design guidelines for acoustic anomaly detection under realistic submarine constraints. Rather than focusing solely on algorithmic performance, the proposed approach integrates proxy data generation, noise modeling, and lightweight model evaluation to derive practical design guidelines for anomaly detection systems deployable in resource-constrained submarine platforms.

## 2. Proxy Data for Rotating Machinery and Environment Noise

**Figure 1:**
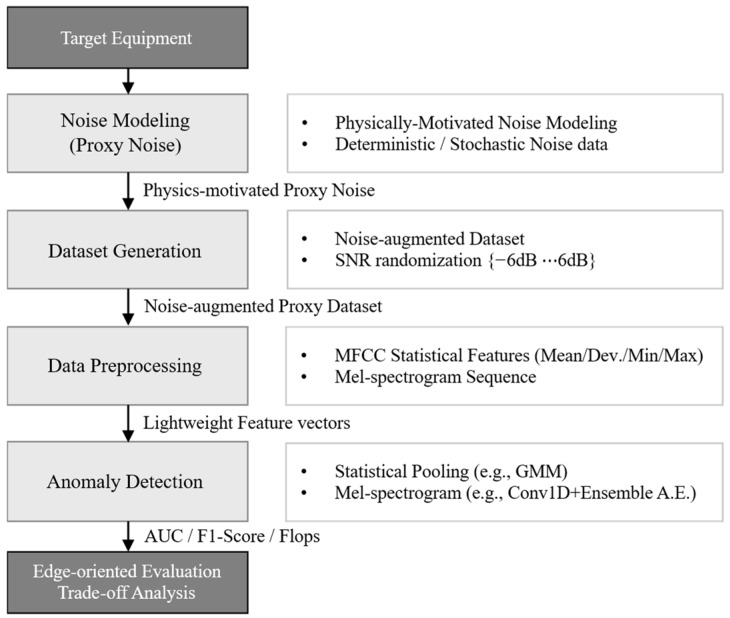
Overall workflow of the proposed acoustic anomaly detection framework under data scarcity and edge computing constraints.

As shown in Figure 1, the overall conceptual framework of the proposed unsupervised learning-based acoustic anomaly detection procedure for submarine onboard equipment consists of sequential stages, including target equipment definition, proxy noise modeling, dataset generation, data preprocessing, anomaly detection, and edge-oriented evaluation with trade-off analysis. First, representative noise components observed in submarine operational environments are modeled to construct proxy acoustic data, which are combined under controlled SNR conditions to generate noise-augmented datasets. Next, the generated data are then processed through a lightweight preprocessing pipeline designed for resource-constrained environments. Finally, multiple unsupervised anomaly detection models are evaluated in terms of both detection performance and computational efficiency to assess their suitability for edge deployment.

### 2.1. Target Equipment and Proxy Data

**Figure 2:**
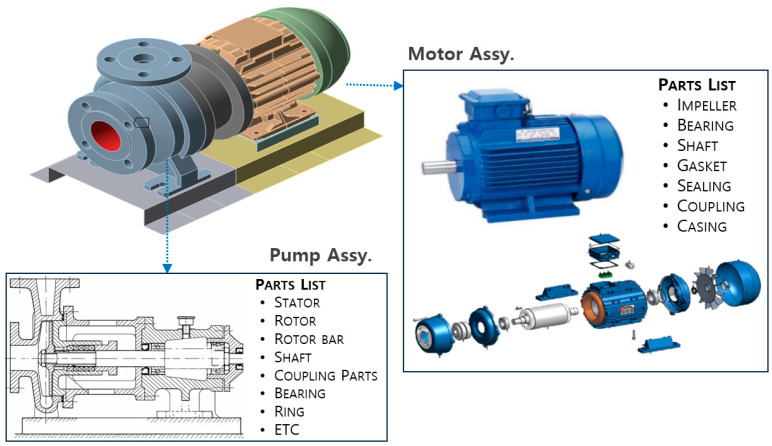
Configuration of Centrifugal Pump (conceptual example).

Submarines operate in highly classified security environments, which makes it difficult to access and utilize actual fault data in open research. Additionally, implementing rigorous preventive maintenance policies minimizes the occurrence of in-service failures, resulting in a scarcity of labeled faults and abnormal data. These constraints hinder the application of supervised learning approaches, making unsupervised anomaly detection based primarily on normal data a more realistic alternative. This study aims to develop an acoustic anomaly detection procedure for submarine onboard rotating machinery, including pump systems (Figure 2). To address the lack of real measurement data, the publicly available MIMII dataset [25] is adopted as a proxy data platform. It should be emphasized that MIMII does not directly represent actual submarine equipment faults; rather, it is used solely as a surrogate platform that preserves the general time–frequency structure and statistical characteristics of rotating machinery acoustic signals. Therefore, the use of MIMII in this study should be interpreted as a proxy data strategy for preliminary methodological validation rather than as direct experimental validation using operational submarine equipment data. Subsequently, noise components that emulate submarine operational environments are synthetically injected into the rotating machinery signals of MIMII to construct training and evaluation datasets under various SNR conditions.

Acoustic sensing can relatively alleviate constraints related to sensor installation, wiring, and maintenance in onboard environments, enabling data collection even within limited physical space. Furthermore, common degradation phenomena in rotating machinery—such as increased bearing friction, valve leakage, and misalignment—are reflected in changes in the energy distribution and spectral characteristics of acoustic signals. Therefore, acoustic signals provide meaningful observational cues for unsupervised anomaly detection [21]. However, it should be noted that the objective of this study is not to demonstrate absolute diagnostic performance for a specific system, but rather to validate the feasibility of anomaly detection procedures under data-scarce conditions and to assess their deployability in resource-constrained edge environments.

The MIMII dataset provides acoustic signals sampled at 16 kHz, each segmented into 10-s recordings. In this study, the pump subset corresponding to model IDs {00, 02, 04, 06} was utilized (Table 2). This subset consists of 3749 normal samples and 456 abnormal samples, totaling 4205 segments [23]. It is important to clarify that the abnormal samples do not constitute diagnostic labels that correspond uniquely to specific fault mechanisms. Rather, they represent abnormal operating or fault conditions characterized by deviations in acoustic patterns from the normal state. The detailed segment composition of the pump subset used in this study is summarized in Table 2.

### 2.2. Definition of Environment Noise Components

Acoustic signals measured within submarines are typically observed as a superposition of multiple interference sources rather than emissions from a single piece of equipment. Furthermore, the noise distribution may vary depending on operational conditions [7,21,26,31]. To reflect these characteristics, this study categorizes dominant noise components in submarine operational environments based on their physical properties as follows: (1) colored-noise-based background noise, (2) structure-borne noise incorporating structural resonance, (3) band-limited auxiliary machinery noise, (4) narrowband tonal components generated by rotating machinery, and (5) sensor measurement noise. These components are not intended to represent specific fault modes but are introduced to construct physically plausible noise conditions. These noise components exhibit distinct statistical and spectral characteristics, making simple white-noise approximation inadequate [32,33].

In this study, each noise component is individually modeled to reflect its physical characteristics and then linearly combined with the original signal to construct a noise-augmented dataset. The combined signal is subsequently scaled to satisfy a target SNR, representing conditions where equipment-generated signals are partially masked by environmental noise. During training, SNR values ranging from −6 dB to 6 dB are randomly applied at the segment level to promote robustness against varying noise intensities. In contrast, fixed SNR conditions (e.g., 0 dB and −6 dB) are used during testing to evaluate anomaly detection performance under more challenging scenarios.

**Figure 3:**
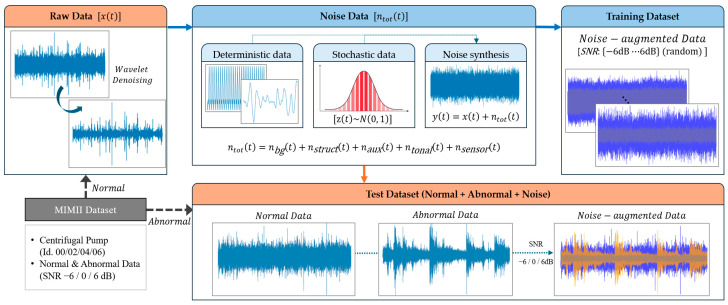
Procedure for physics-motivated noise synthesis and SNR-controlled augmentation of the MIMII proxy dataset for anomaly detection.

As illustrated in Figure 3, the clean signal x(t) is combined with multiple noise components—including background noise $n_{bg}(t)$, structure-borne noise $n_{struct}(t)$, auxiliary noise $n_{aux}(t)$, tonal components $n_{tonal}(t)$, and sensor noise $n_{sensor}(t)$—to form the total noise $n_{tot}(t)$. The final noise-augmented signal is obtained through linear superposition as $y(t)=x(t)+n_{tot}(t)$, followed by scaling to meet the target SNR. This data construction strategy emulates realistic operational environments in which multiple noise sources coexist simultaneously and enables evaluation of the robustness of unsupervised anomaly detection algorithms under varying noise conditions.

#### 2.2.1. Background Noise (Ambient Noise)

**Figure 4:**
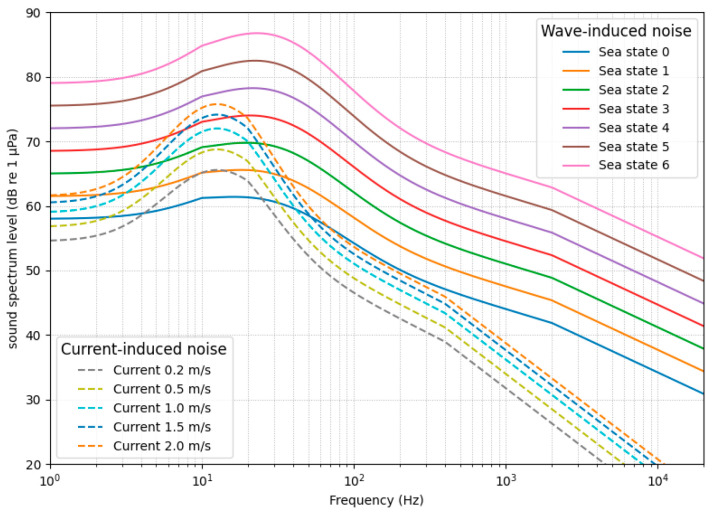
Conceptual characterization of ocean background noise: frequency-domain noise curves under waves and current conditions.

Acoustic signals measured within submarines are typically observed as superpositions that include stochastic background noise originating from the operational environment [31,34]. Such background noise is generally characterized as a non-periodic random signal without distinct narrowband structures. The spectral characteristics of marine ambient noise, commonly represented by Wenz curves, exhibit dominant energy in the low-frequency band with gradual decay as frequency increases (Figure 4) [31,35]. To capture this behavior, background noise is modeled as colored noise with a power spectral density (PSD) following $S_{bg}(f)\propto 1/f^{\alpha }\left(\alpha >0\right)$ [7,34], where α controls the low-frequency dominance. In this study, the spectral exponent is defined within the range 1.0≤α≤2.0 to represent typical marine ambient noise characteristics.

The background noise $n_{bg}(t)$ was generated in the frequency domain by applying spectral shaping proportional to $1/f^{\alpha /2}$, resulting in a PSD of $S_{bg}(f)\propto 1/f^{\alpha }$. The generated noise was linearly superimposed on the original signal x(t), and its magnitude was adjusted to satisfy a predefined target SNR. The SNR was defined as $\mathrm{SNR}\;(\gamma )\approx 10{log}_{10}(P_{x}/P_{n})$, where $P_{x}$ and $P_{n}$ denote the average power of the signal and noise, respectively [26]. The scaling coefficient a was computed to meet the target SNR as Equation (1) [7], and the final background noise was obtained as Equation (2). The spectral exponent α was randomly sampled from a uniform distribution within the range 1.0≤α≤2.0, and the remaining parameters are summarized in Table 3.

(1) $$a=\sqrt{\frac{P_{x}}{P_{\tilde{n}}\cdot 10^{\gamma /10}}}$$

(2) $$n_{bg}\left(t\right)=a\;{\tilde{n}}_{bg}\left(t\right)$$

**Figure 5:**
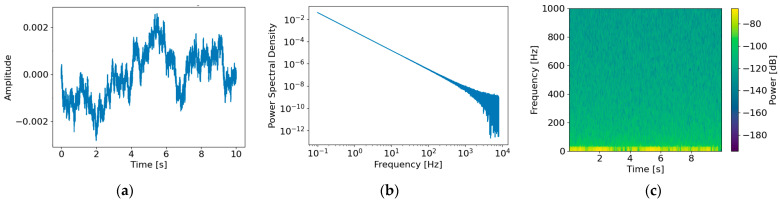
Characteristics of the generated background noise $n_{bg}\left(t\right)$: (**a**) time-domain waveform, (**b**) power spectral density in the frequency domain, and (**c**) time–frequency spectrogram.

Figure 5 presents the time–frequency characteristics of the generated background noise $n_{bg}(t)$ under an SNR of 0 dB. The time-domain waveform (Figure 5a) exhibits non-periodic stochastic behavior, while the PSD (Figure 5b) shows dominant energy in the low-frequency band with gradual decay as frequency increases, consistent with the characteristics of marine ambient noise. The spectrogram (Figure 5c) further confirms broadband energy distribution without distinct narrowband components, indicating that the generated noise appropriately reflects colored-noise characteristics.

#### 2.2.2. Modeling of Structure-Borne Noise

Structure-borne noise refers to vibration-induced noise transmitted through structural components and added to the measured signal. Rather than constructing a high-fidelity dynamic model, this study adopts a simplified representation in which the structural transmission path is modeled as a linear time-invariant (LTI) system characterized by its frequency response [36,37]. Accordingly, the structure-borne noise $n_{struct}(t)$ is modeled as the output of a filtering process in which an input noise sequence ${\tilde{n}}_{struct}(t)$ passes through the structural impulse response $h_{struct}(t)$:

(3) $$n_{struct}(t)=h_{struct}(t)*{\tilde{n}}_{struct}(t)$$

Here, ${\tilde{n}}_{struct}(t)$ denotes zero-mean white Gaussian noise, and $h_{struct}(t)$ represents a conceptual impulse response capturing resonance characteristics. The corresponding frequency response H(f) is defined as a spectral shaping function to represent selective amplification caused by structural resonances. To approximate multi-degree-of-freedom resonance behavior, H(f) is modeled as a superposition of K Gaussian peaks:

(4) $$H\left(f\right)=1+\sum_{k=1}^{K}g_{k}\exp\left(-\frac{{\left(f-f_{0,k}\right)}^{2}}{2\;bw_{k}^{2}}\right)$$

Here, $f_{0,k}$ denotes the k-th resonance frequency corresponding to structural vibration modes associated with internal hull components such as frames and bulkheads. In this study, K=3 is used to represent dominant low- to mid-frequency structural resonances. The resonance frequencies $f_{0,k}$ are defined within the range of 50–500 Hz, with representative values of 80 Hz, 180 Hz, and 420 Hz. The gain coefficient $g_{k}$ controls the amplification level (approximately 3–6 dB), and is defined as $g_{k}=10^{G_{dB}/20}-1$. The bandwidth parameter $bw_{k}$ is set to 10 Hz to represent moderate resonance effects under structural damping conditions. The parameters used in the structure-borne noise generation process are summarized in Table 4. This formulation is intended to capture representative resonance amplification behavior rather than to model exact structural dynamics of a specific submarine configuration [32].

**Figure 6:**
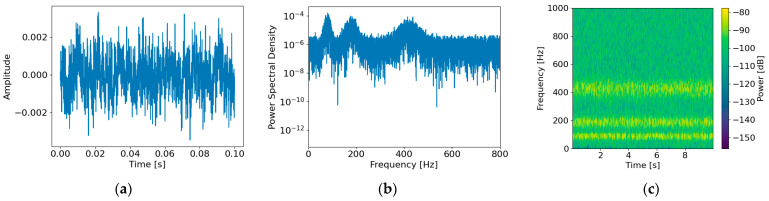
Characteristics of the generated structure-borne noise $n_{struct}\left(t\right)$: (**a**) time-domain waveform, (**b**) power spectral density, and (**c**) time–frequency spectrogram.

Figure 6 illustrates the time–frequency domain characteristics of the generated structure-borne noise $n_{struct}(t)$ under an SNR of 0 dB. In the time domain (Figure 6a), the signal exhibits continuous oscillatory behavior rather than purely random fluctuations, reflecting the influence of structural dynamic response. The time–frequency spectrogram (Figure 6c) shows sustained energy concentration around specific resonance bands over time, while other frequency regions exhibit relatively low energy. These results confirm that the modeled structure-borne noise differs from white noise with uniformly distributed spectral energy and instead represents a non-white interference component shaped by structural resonance effects.

#### 2.2.3. Auxiliary Noise (Band-Limited Broadband)

Auxiliary noise refers to mechanical noise components continuously generated by onboard subsystems, such as air-conditioning, cooling, and circulation systems in submarines. This type of noise fluctuates irregularly over time depending on equipment operating conditions and fluid flow characteristics. In the frequency domain, it exhibits broadband characteristics with energy distributed over a finite frequency range rather than being confined to discrete frequencies [33,38]. In particular, noise generated by pump and cooling systems is typically induced by fluid turbulence and pressure fluctuations, resulting in band-limited colored noise spanning the low- to mid-frequency range. Broadband mechanical noise is generally modeled as a stochastic process and is often assumed to be a zero-mean stationary random signal characterized by its statistical properties. However, due to the practical difficulty of separately measuring auxiliary noise from individual onboard subsystems in real submarine environments, this study adopts a simplified probabilistic modeling approach (Equation (5)). Specifically, auxiliary noise is modeled by passing a zero-mean Gaussian white noise sequence ${\tilde{n}}_{aux}(t)$ through a band-limited filter $h_{aux}(t)$ that reflects the frequency characteristics of auxiliary machinery noise.

(5) $$n_{aux}\left(t\right)=h_{aux}\left(t\right)*{\tilde{n}}_{aux}\left(t\right)$$

Here, $h_{aux}(t)$ represents a conceptual transfer function that approximates the band-limited spectral characteristics of auxiliary subsystems. In this study, $h_{aux}(t)$ is defined as a spectral shaping filter, and the auxiliary noise $n_{aux}(t)$ is generated by filtering a white Gaussian noise sequence ${\tilde{n}}_{aux}(t)$. The resulting power spectral density (PSD) is determined by the imposed frequency response and satisfies the predefined target PSD (Equation (6)).

(6) $$S_{n_{aux}}\left(f\right)=|H_{aux}\left(f\right){|}^{2}S_{\tilde{n}}\left(f\right)$$

To clarify the parameter settings used in the auxiliary noise generation process, the key parameters of the band-limited filter are summarized in Table 5. In this study, auxiliary noise is generated by filtering a Gaussian noise sequence through a band-limited filter designed to approximate the spectral characteristics of auxiliary mechanical subsystems. The lower cutoff frequency $f_{low}$ is set to 20 Hz to exclude very low-frequency drift components, while the upper cutoff frequency $f_{high}$ is set to 1.5 kHz to reflect the dominant low- to mid-frequency characteristics of auxiliary equipment noise [33,39]. This band-limited filtering enables the generated noise to exhibit broadband stochastic behavior consistent with auxiliary mechanical subsystems.

**Figure 7:**
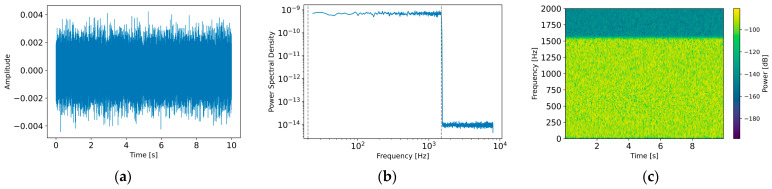
Characteristics of the generated auxiliary noise $n_{aux}\left(t\right)$: (**a**) time-domain waveform, (**b**) power spectral density in the frequency domain, and (**c**) time–frequency spectrogram.

Figure 7 illustrates the time–frequency domain characteristics of the generated auxiliary noise $n_{aux}(t)$ under an SNR of 0 dB. In the time domain (Figure 7a), the signal exhibits stochastic behavior without distinct periodicity or repetitive structures, reflecting continuous mechanical noise from auxiliary subsystems. In the frequency domain (Figure 7b), the noise shows broadband characteristics with energy distributed across the low- to mid-frequency range rather than being concentrated at discrete frequencies. A clear band-limited characteristic is also observed, with the PSD sharply decreasing beyond the predefined frequency range. The time–frequency spectrogram (Figure 7c) further shows relatively uniform energy distribution over time within this band. These results confirm that the auxiliary noise is modeled as stochastic broadband noise rather than as deterministic periodic components such as tonal signals.

#### 2.2.4. Tonal Component of Rotating Machinery

The tonal component represents narrowband spectral components generated by the periodic motion of rotating machinery and is typically observed as line-spectrum components at discrete frequencies determined by the rotational speed. Unlike auxiliary or background noise, tonal components exhibit periodic behavior in the time domain, while their energy is concentrated at the fundamental frequency and its harmonics in the frequency domain. The fundamental frequency $f_{0}$ is defined by the rotational speed (RPM) as $f_{0}=\mathrm{RPM}/60$.

In practical measurements, both the fundamental frequency $f_{0}$ and its harmonic components $f_{k}=kf_{0}$ are commonly observed due to nonlinearities and mechanical characteristics of rotating systems. In this formulation, k denotes the harmonic order (i.e., the maximum harmonic index considered). Although harmonic components can be associated with abnormal behavior [21], they may also arise under normal operating conditions due to factors such as shaft asymmetry and structural non-uniformities [40,41]. In this study, the tonal component is modeled as a deterministic signal rather than stochastic noise, reflecting its stable periodic behavior and frequency-dependent structure. Accordingly, the tonal component $n_{tonal}(t)$ is defined as a linear combination of the fundamental frequency and a finite number of harmonic components, as expressed in Equation (7).

(7) $$n_{tonal}\left(t\right)=\sum_{k=1}^{K}A_{k}cos\left(2\pi kf_{0}t+\phi_{k}\right)$$

Here, $A_{k}$ and $\phi_{k}$ denote the amplitude and initial phase of the k-th harmonic component, respectively, and K represents the maximum harmonic order. The harmonic order is constrained such that the harmonic frequencies do not exceed the Nyquist frequency. Given the sampling frequency $f_{s}=16\;\mathrm{kHz}$, the Nyquist frequency is defined as $f_{N}=f_{s}/2$, and only harmonics satisfying $kf_{0}\leq f_{N}$ are considered. Since the objective of this study is to represent characteristic line-spectrum structures, rather than detailed dynamic behavior, the harmonic order is practically limited to lower-order components. Previous studies indicate that dominant spectral energy is concentrated within the first few harmonics, typically up to the 3rd–5th orders, while higher-order components tend to attenuate due to structural damping and transmission losses [21,41]. Accordingly, the harmonic order K is limited to 5. Table 6 summarizes the parameters used in tonal component generation. To reflect normal operating variability, the rotational speed is assumed to range from 900 to 1800 RPM.

**Figure 8:**
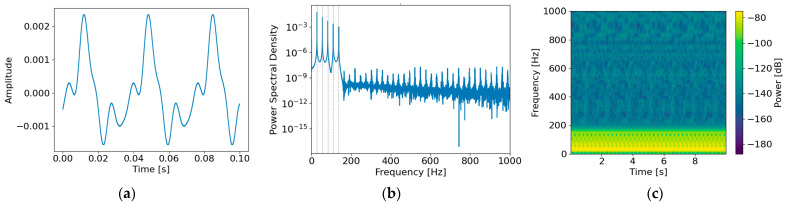
Characteristics of the generated tonal noise $n_{tonal}\left(t\right)$: (**a**) time-domain waveform, (**b**) power spectral density in the frequency domain, and (**c**) time–frequency spectrogram.

Figure 8 illustrates the time–frequency domain characteristics of the generated rotational tonal component $n_{tonal}(t)$ under an SNR of 0 dB. In the time domain (Figure 8a), the tonal component exhibits a periodic waveform with stable amplitude, reflecting deterministic behavior induced by repetitive rotational motion. In the frequency domain (Figure 8b), a distinct line-spectrum peak appears at the fundamental frequency $f_{0}\approx 27.5\;\mathrm{Hz}$ (corresponding to approximately 1650 RPM), along with harmonic peaks at k=2,3,4,5. In the time–frequency spectrogram (Figure 8c), both the fundamental and harmonic components appear as continuous line structures over time. These results confirm that the tonal component is modeled as a deterministic signal rather than stochastic noise.

#### 2.2.5. Sensor Noise (Measurement Noise)

Sensor noise refers to noise components generated within the measurement system itself, originating from sources such as electronic circuitry and analog-to-digital (A/D) conversion processes. Unlike environmental or equipment-induced noise, sensor noise is not directly associated with the operational state of the target equipment and is inherently present throughout the measurement process [26,37]. In the frequency domain, it typically exhibits a flat spectral distribution, resembling white noise characteristics [7]. In this study, sensor noise is modeled as Gaussian white noise, as defined in Equation (8), where the noise magnitude is controlled by a scaling coefficient $\sigma_{s}$. The noise sequence $\tilde{n}$_sensor (t) is assumed to follow a standard normal distribution, ${\tilde{n}}_{sensor}\left(t\right)\sim N(0,1)$:

(8) $$n_{sensor}\left(t\right)=\sigma_{s}\;{\tilde{n}}_{sensor}\left(t\right)$$

Accordingly, the sensor noise has zero mean, $E[n_{sensor}(t)]=0$, and a constant PSD $S_{n_{sensor}}(f)=\sigma_{s}^{2}$ across the entire frequency band. Table 7 summarizes the parameters used in the sensor noise generation model.

**Figure 9:**
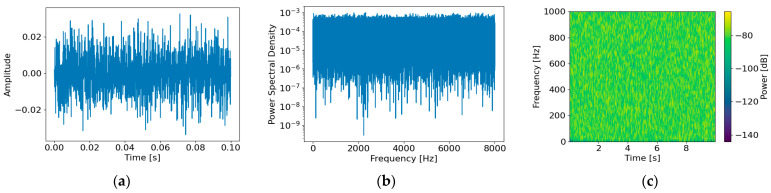
Characteristics of the generated sensor noise $n_{sensor}\left(t\right)$: (**a**) time-domain waveform, (**b**) power spectral density in the frequency domain, and (**c**) time–frequency spectrogram.

Figure 9 illustrates the statistical characteristics of the generated sensor noise $n_{sensor}(t)$ under an SNR of 0 dB. In the time domain (Figure 9a), the waveform exhibits irregular fluctuations without distinct periodicity, confirming the stochastic nature of the sensor noise. In the frequency domain (Figure 9b), the power spectrum shows a flat energy distribution across the entire frequency range, consistent with white noise characteristics. The time–frequency spectrogram (Figure 9c) further demonstrates a uniform energy distribution over frequency, validating that the sensor noise is appropriately modeled as Gaussian white noise.

### 2.3. Noise-Augmented Dataset of Rotating Machinery

In this study, a noise-augmented dataset was constructed by superimposing multiple noise components—$n_{bg}(t)$, $n_{struct}(t)$, $n_{aux}(t)$, $n_{tonal}(t)$, and $n_{sensor}(t)$—onto the clean signal x(t), as expressed in Equations (9) and (10), where $n_{tot}(t)$ denotes the composite noise.

(9) $$y\left(t\right)=x\left(t\right)+a\cdot n_{tot}\left(t\right)$$

(10) $$n_{tot}\left(t\right)=n_{\mathrm{bg}}\left(t\right)+n_{\mathrm{struct}}\left(t\right)+n_{\mathrm{aux}}\left(t\right)+n_{\mathrm{tonal}}\left(t\right)+n_{\mathrm{sensor}}\left(t\right)$$

Since direct summation increases the overall noise power and can excessively degrade the SNR, a global scaling coefficient α is applied to the composite noise to achieve a predefined target SNR. The target SNR is defined as ${\mathrm{SNR}}_{\mathrm{target}}(\mathrm{dB})=10{log}_{10}(P_{x}/P_{n_{tot}})$, where $P_{x}$ and $P_{n_{tot}}$ denote the average power of the clean signal and total noise, respectively. The scaling coefficient is computed as:

(11) $$\alpha =\sqrt{\frac{P_{x}}{P_{n_{t}ot}\cdot 10^{{SNR}_{target}/10}}}$$

(12) $$P_{n_{tot}}={\mathrm{RMS}}^{2}\left(n_{tot}\right)$$

(13) $$\mathrm{RMS}\left(n_{tot}\right)=\sqrt{\frac{1}{N}\sum_{i=1}^{N}n_{tot}^{2}\left(i\right)}$$

**Figure 10:**
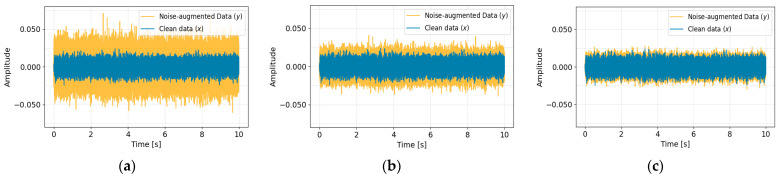
Time-domain comparison between clean data and noise-augmented data under different SNR conditions: (**a**) SNR −6 dB, (**b**) SNR 0 dB, and (**c**) SNR 6 dB.

**Figure 11:**
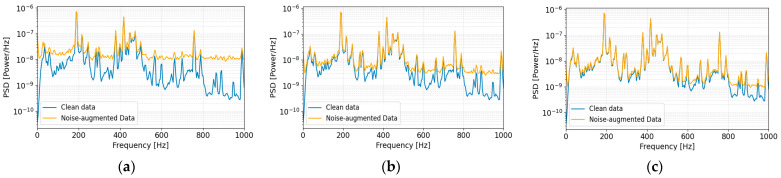
Power spectral density comparison between clean data and noise-augmented data under different SNR conditions: (**a**) SNR −6 dB, (**b**) SNR 0 dB, and (**c**) SNR 6 dB.

Figure 10 compares the clean signal with the noise-augmented signal under SNR conditions of −6 dB, 0 dB, and 6 dB. The PSD results in Figure 11 show that the augmented signals preserve the overall spectral structure while increasing energy levels, particularly in the low- to mid-frequency range due to the superposition of multiple noise components. As the noise level increases, energy rises consistently without introducing artificial spectral peaks or impulsive components, indicating that the synthesized signals realistically emulate environmental noise effects.

The dataset is generated using a physics-motivated model; therefore, the evaluation focuses on generalization rather than exact reproduction of a specific environment. To reflect variability in operating conditions, key parameters—including the spectral exponent α, structural resonance parameters, band-limited filter ranges, and rotational speed (RPM)—are randomized during training. The selected SNR levels (−6 dB, 0 dB, and 6 dB) represent practical noise conditions and enable quantitative evaluation of algorithm robustness.

## 3. Anomaly Detection Based on Statistical Features

This section presents the construction of statistical representations of the input data, which are subsequently utilized as training features for unsupervised anomaly detection. Two representative modeling approaches are considered: the probabilistic GMM and a reconstruction-error-based Ensemble Autoencoder. All models are trained using the noise-augmented proxy dataset derived from the MIMII dataset.

### 3.1. Data Preprocessing to Extract Statistical Features

**Figure 12:**
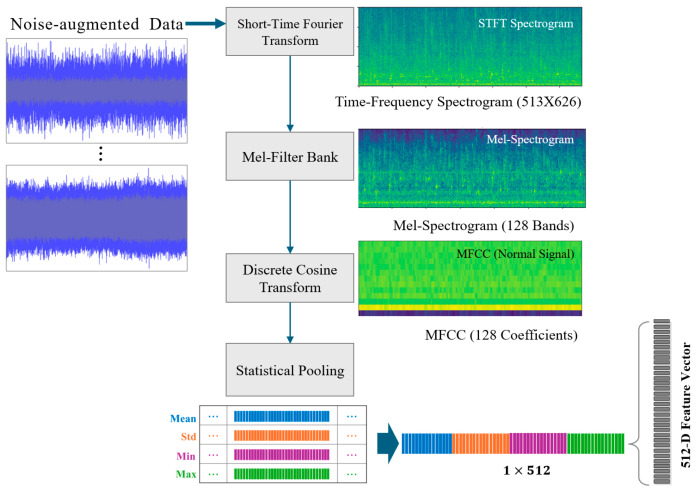
Feature extraction pipeline for statistical pooling of MFCCs into a 512-dimensional feature vector.

During data preprocessing, time-series characteristics were analyzed using the Short-Time Fourier Transform (STFT) and MFCCs [42,43]. To ensure real-time applicability, the preprocessing pipeline was designed with an emphasis on lightweight feature representation for anomaly detection. First, the STFT was applied to compute the time–frequency spectrum, converting each raw signal into a two-dimensional matrix of size 513 × 626. Subsequently, a Mel filter bank and Discrete Cosine Transform (DCT) were employed to extract 128-dimensional MFCCs, capturing perceptually relevant frequency sensitivity and spectral variation patterns. To further reduce input dimensionality while preserving discriminative information, four statistical features—mean, standard deviation, minimum, and maximum—were computed for each MFCC. This process transformed the original 128 × 4 representation into a fixed-length 512-dimensional feature vector. By summarizing temporal information through statistical pooling, redundant time-axis information was suppressed while retaining distributional characteristics sensitive to abnormal patterns (Figure 12). The resulting 512-dimensional feature vectors were subsequently normalized using a StandardScaler(scikit-learn v1.5.1) to standardize feature distributions prior to input into the anomaly detection models. This normalization step prevents disproportionate weighting of specific features during training, ensures consistent scaling across all dimensions, and contributes to improved computational efficiency.

### 3.2. Anomaly Detection Using GMM

**Figure 13:**
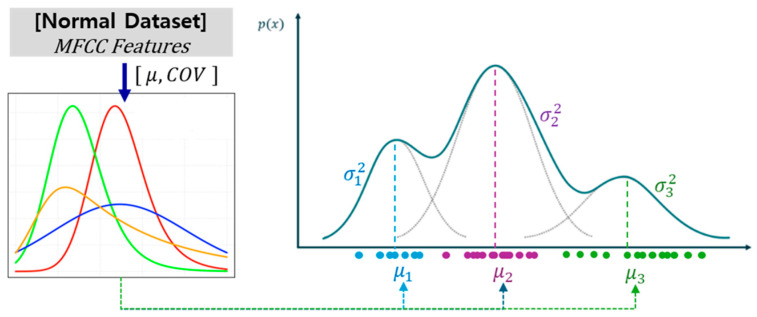
The Gaussian Mixture Model with multiple Gaussian distributions.

The GMM is a probabilistic framework that models the distribution of normal data as a linear combination of multiple Gaussian components. This formulation enables flexible representation of complex normal-data distributions that cannot be adequately described by a single unimodal Gaussian distribution, thereby allowing more adaptive decision boundaries for anomaly detection [44,45]. Table 8 summarizes the principal hyperparameters of the GMM algorithm and the corresponding search space defined for grid search optimization. As a probability density-based model, the GMM represents the data distribution as a mixture of multivariate normal distributions. Among its hyperparameters, the number of mixture components ($n_{\mathrm{components}}$) and the covariance structure (covariance_type) directly influence both the representational capacity and computational complexity of the model. Furthermore, the covariance regularization term (reg_covar) serves as a numerical stabilization parameter, preventing overfitting caused by excessively small variance estimates. In this study, the optimal value of $n_{\mathrm{components}}$ was determined through grid search using the Bayesian Information Criterion (BIC) as the model selection criterion. Candidate values within a predefined search space were evaluated for each dataset, and the value minimizing the BIC score was selected as the optimal number of mixture components. The probability density function of the GMM is defined as shown in Equation (14) (Figure 13).

(14) $$P_{\left(x\right)}=\sum_{k=1}^{K}\pi_{k}N\left(x|\mu_{k},\;\sum k\right)$$

In Equation (14), K denotes the number of mixture components, $\pi_{k}$ represents the mixture weight of the k-th component, and $\mu_{k}$ and $\Sigma_{k}$ correspond to the mean vector and covariance matrix, respectively. During training, the GMM parameters are estimated using the Expectation–Maximization (EM) algorithm. In the E-step, the posterior probability (responsibility) of a sample $x_{i}$ belonging to each Gaussian component is computed. During the M-step, the mixture weights, mean vectors, and covariance matrices are updated accordingly. This iterative procedure continues until the log-likelihood converges, resulting in a probabilistic model that effectively characterizes the distribution of normal data.

After training the model on normal data, the log-likelihood of a new input sample x is computed as defined in Equation (15). The log-likelihood value quantifies the probabilistic confidence that the sample belongs to the learned normal distribution. A decision threshold τ is determined from the log-likelihood distribution of the normal training data by selecting the lower 5th percentile, as shown in Equation (16). Based on this threshold, a sample is classified as normal if ℓ(x)≥τ and as anomalous if ℓ(x)<τ.

(15) $$\ell \left(x\right)=logp\left(x\right)$$

(16) $$\tau ={quantile(\{\ell (x_{i})\}}_{x_{i}\in Normal},0.05)$$

### 3.3. Anomaly Detection Using Ensemble Autoencoder

An Autoencoder is an unsupervised neural network that learns to compress input data into a low-dimensional latent space and subsequently reconstruct it. It consists of an encoder, which maps unlabeled training data into a latent representation, and a decoder, which reconstructs the input from this internal representation [46,47]. Since an Autoencoder is trained to minimize reconstruction error for normal data, it does not explicitly learn a decision boundary separating normal and abnormal samples. Consequently, anomalous data with patterns resembling those of normal data may yield low reconstruction error, potentially degrading anomaly detection performance. To address this limitation, the present study employs an Ensemble Autoencoder architecture. This ensemble consists of multiple Autoencoders with different latent dimensions trained in parallel, with anomaly decisions made by combining the reconstruction errors from each model.

**Figure 14:**
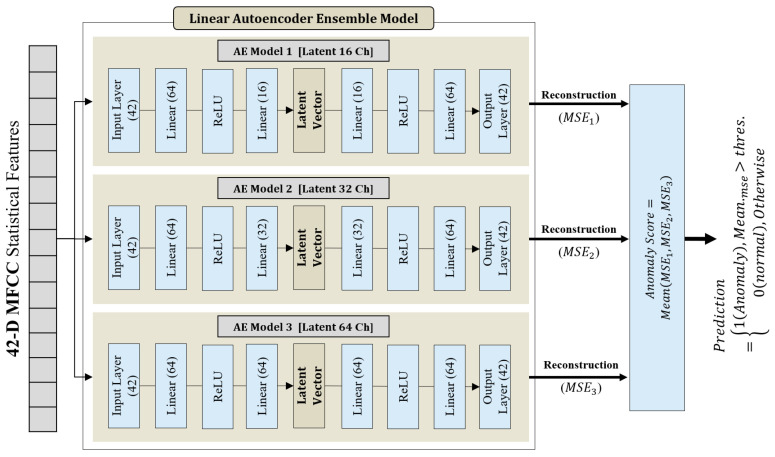
Architecture of the Ensemble Autoencoder-based Anomaly Detection Model.

Figure 14 illustrates the architecture of the Linear Ensemble Autoencoder-based anomaly detection model. In the GMM-based approach, a high-dimensional statistical feature vector was constructed to more precisely represent the density structure of the normal data distribution. Specifically, four statistical measures—mean, standard deviation, minimum, and maximum—were extracted for the 128 MFCCs, yielding a 512-dimensional feature vector that served as the model input. In contrast, for the Autoencoder-based model, the input dimensionality was reduced to 42 dimensions to improve computational efficiency and generalization performance. More specifically, only the lower-order 42 MFCCs were selected to construct the statistical feature vector. Low-order MFCCs primarily capture the overall spectral envelope and dominant frequency structure of acoustic signals, whereas higher-order coefficients tend to reflect fine spectral variations and noise-sensitive components. From an anomaly detection perspective, the principal characteristics of normal operating conditions can be effectively represented using only the low-order coefficients [48]. Reducing the input dimensionality decreased the number of trainable parameters in the Autoencoder, thereby lowering computational complexity and memory usage during both training and inference. This dimensionality reduction also helps mitigate overfitting, particularly in data-limited environments where excessive model capacity may degrade generalization performance. Based on this lightweight feature representation, three independent Linear Autoencoders with distinct latent dimensions were constructed in parallel to form an ensemble structure.

Each Autoencoder is trained independently using only normal data, with the reconstruction loss computed as the Mean Squared Error (MSE) between the input and the reconstructed output. During the anomaly detection phase, the reconstruction errors obtained from each Autoencoder, denoted as ${\mathrm{MSE}}_{1}$, ${\mathrm{MSE}}_{2}$, and ${\mathrm{MSE}}_{3}$, are combined to produce a final anomaly score. In this study, the final anomaly score is computed as the arithmetic mean of the reconstruction errors across the ensemble, thereby mitigating the influence of extreme error values that may arise from a specific model. Let N denote the number of samples, $x_{i}$ represent the i-th input sample, and ${\hat{x}}_{i}^{\left(k\right)}$ denote its reconstruction from the k-th Autoencoder. The reconstruction error is defined as shown in Equation (17) [49].

(17) $$\mathrm{Reconstruction}\;\mathrm{Loss}=\frac{1}{N}\sum_{i=1}^{N}\left.|x_{i}-\hat{x_{i}}\right.{|}^{2}$$

Table 9 summarizes the principal hyperparameters of the Ensemble Autoencoder-based anomaly detection model. The ensemble architecture comprises Autoencoders with different latent dimensions (16, 32, and 64), which were selected to mitigate bias arising from a single latent-space configuration and to enable more effective representation of diverse normal operating patterns. Model training was performed in an unsupervised manner using only normal data. The learning rate was fixed at $1\times 10^{-3}$, the batch size was set to 32, and the number of training epochs was 50. Parameter optimization was conducted using the Adam optimizer, and the Mean Squared Error (MSE) between the input and reconstructed output was used as the reconstruction loss function. For anomaly detection, a decision threshold was established based on the distribution of reconstruction errors obtained from the normal training data. Specifically, the threshold was set at the 95th percentile (i.e., the upper 5%) of the reconstruction error distribution computed from the normal training data. During testing, samples whose reconstruction error exceeded this threshold were classified as anomalous. This threshold was adopted as a baseline criterion for comparative evaluation rather than as an optimal operational setting.

## 4. Anomaly Detection Based on Data-Windowing

To address the limitations observed in the statistical feature-based anomaly detection approach described in the previous section, a data-windowing-based input representation combined with a Conv1D–Ensemble Autoencoder is introduced in this section. The proposed method preserves the temporal characteristics of the time–frequency structure of acoustic signals, thereby enabling more effective emphasis on localized anomalous patterns that may otherwise be obscured when global statistical features are employed.

### 4.1. Data Preprocessing for Data-Windowing

All input signals were uniformly resampled at a sampling frequency of 16 kHz and included environmental noise components. For each acoustic signal, a Mel-spectrogram was computed based on the STFT. The number of Mel filter banks was set to 128, with an FFT size of 2048 and a hop length of 512. A logarithmic transformation was then applied to the power spectrogram to generate a log Mel-spectrogram representation. The resulting log Mel-spectrogram was segmented along the time axis using a fixed-length sliding window.

In this study, each window comprised 32 frames, corresponding to approximately one second of audio. To incorporate overlap between adjacent segments, the stride was set to 16 frames, resulting in a 50% overlap. This windowing strategy generated multiple temporally localized samples from a single acoustic signal, thereby enabling short-duration anomalous patterns to be captured as independent training samples. When the spectrogram length was shorter than the predefined window size, zero-padding was applied to maintain a consistent input dimension. After windowing, standardization was performed along the Mel-filter dimension for all generated samples to prevent specific frequency bands from exerting disproportionate influence during training.

### 4.2. Conv1D–Ensemble Autoencoder Architecture

The anomaly detection model adopts an Ensemble Autoencoder architecture based on a one-dimensional convolutional neural network (Conv1D). Conv1D is effective in extracting localized temporal patterns along the time axis and requires lower computational cost than recurrent neural network (RNN)-based models, making it suitable for real-time operational environments [50]. The input data are structured such that the Mel-filter dimension is treated as the channel dimension and the time axis corresponds to the sequence length. Conv1D operations are applied along the temporal direction to learn time-dependent features. The encoder consists of multiple Conv1D–Batch Normalization–ReLU–Pooling blocks, which progressively compress the input signal into a low-dimensional latent representation. The decoder is symmetrically designed using Upsampling and Conv1D layers to reconstruct the input from the latent representation. The detailed hyperparameters and layer-wise structural configuration of the Conv1D-based Ensemble Autoencoder are summarized in Table 10.

**Figure 15:**
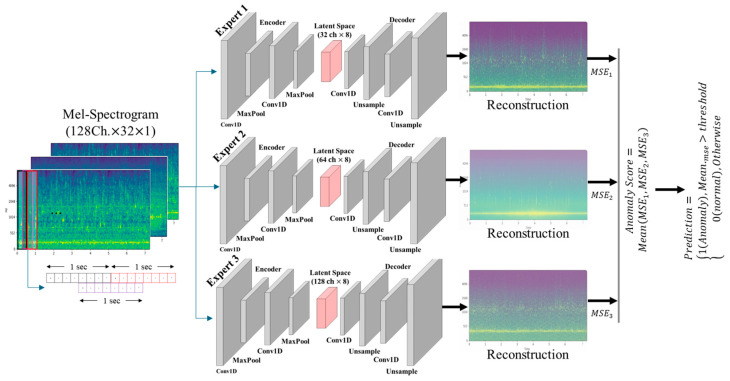
Architecture of the Conv1D-Ensemble Autoencoder model.

To improve model robustness, multiple Conv1D Autoencoders with different latent channel dimensions were constructed in parallel. Specifically, three Conv1D-based Ensemble Autoencoders with latent dimensions of 32, 64, and 128 were employed to facilitate the simultaneous learning of normal acoustic time–frequency patterns across diverse representation spaces. This ensemble configuration mitigates overfitting of individual models to specific noise conditions or equipment characteristics, thereby enhancing overall generalization performance. The final anomaly score is computed as the arithmetic mean of the reconstruction errors across the ensemble models, which minimizes the influence of extreme outputs from individual Autoencoders (Figure 15).

## 5. Comparison of Algorithm Performance Considering the Real-Time Monitoring

In this section, the performance of the proposed anomaly detection algorithms is compared from a real-time operational perspective. Beyond conventional classification performance metrics, the models are comprehensively evaluated in terms of training and inference time, number of parameters, computational complexity (FLOPs), and memory usage to assess their suitability for deployment in embedded or edge computing environments. All experiments were conducted on a Mini-PC platform rather than a high-performance server. Table 11 summarizes the primary specifications of the computing hardware. The selected Mini-PC represents a compact, low-power system that can be deployed within space- and power-constrained environments and is currently integrated with a data acquisition (DAQ) system as part of a prototype platform. It should be noted that the Mini-PC does not directly correspond to the final embedded processor intended for submarine deployment. Instead, it serves as a testbed environment for integrated validation of the full pipeline, including data acquisition, preprocessing, and inference. Training and inference time, as well as memory usage, were measured using CPU-based execution without GPU acceleration.

**Figure 16:**
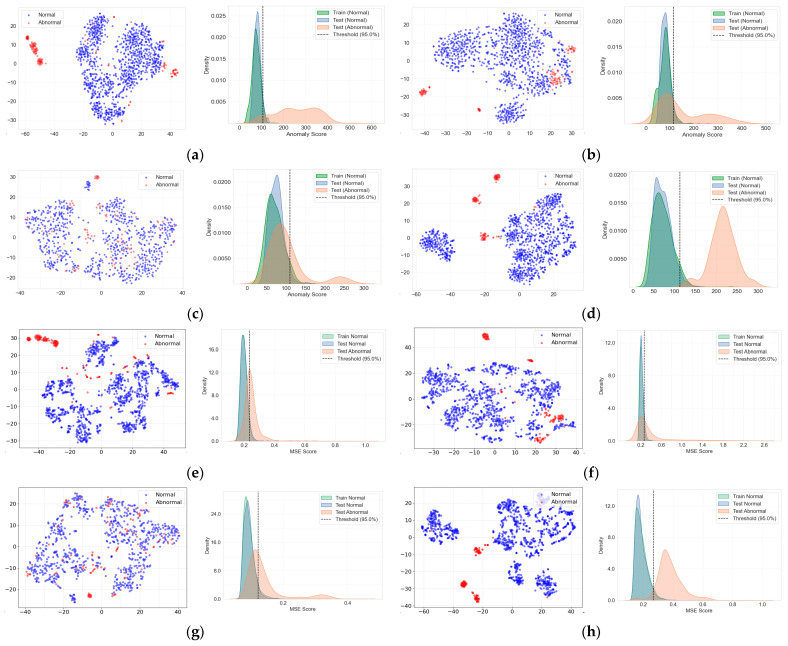
The t-SNE visualizations and KDE plots for different input data: (**a**) GMM-Id.00, (**b**) GMM-Id.02, (**c**) GMM-Id.04, (**d**) GMM-Id.06, (**e**) Conv1D/Ensemble A.E-Id.00, (**f**) Conv1D/Ensemble A.E-Id.02, (**g**) Conv1D/Ensemble A.E-Id.04, and (**h**) Conv1D/Ensemble A.E-Id.06.

Table 12 and Figure 16 present a comprehensive comparison of the performance metrics and data distribution characteristics of the evaluated unsupervised anomaly detection algorithms under noise-augmented conditions. To provide a more comprehensive evaluation of model performance, additional metrics including accuracy and specificity are also reported in Table 12. In addition, all experiments were repeated five times using different random seeds, and the results are presented as the mean and standard deviation (mean ± standard deviation) across the repeated experiments. Overall, under the noise-reflected training condition (Noisy), both the GMM and Autoencoder-based models exhibited improved performance compared to the clean training condition.

For the GMM, the use of noise-augmented data resulted in noticeable performance improvements for Id.00 and Id.06. In addition to the increase in AUC, Precision and Recall also became more balanced (Table 12). This improvement can be attributed to the expanded normal-state distribution induced by environmental noise, which enables the probability density-based GMM to learn a broader representation of the normal data distribution. The t-SNE visualizations further support this observation. For Id.00 and Id.06, normal and abnormal samples form relatively well-separated clusters in the feature space (Figure 16a,d). In contrast, for Id.02 and Id.04, normal and abnormal samples exhibit substantial overlap in the feature space, resulting in relatively limited improvements in F1-score and Recall for the GMM (Figure 16b,c). This overlap indicates that the probabilistic decision boundary learned by the GMM becomes less effective when the class distributions are highly intertwined. The statistical feature-based Ensemble Autoencoder generally achieved higher AUC and F1-scores compared to the GMM, demonstrating particularly stable performance for Id.00 and Id.06. This behavior can be attributed to the Autoencoder’s ability to learn global characteristics of normal patterns through reconstruction error minimization, enabling it to model more complex normal distributions than probability density estimation-based approaches. However, the performance improvements for Id.02 and Id.04 were relatively limited. This can be explained by the fact that the entire acoustic signal is summarized into a single statistical feature vector, where temporally localized anomalous patterns may be diluted during the aggregation process.

In contrast, the Conv1D-based Ensemble Autoencoder, which uses 1-s sliced Mel-spectrogram segments as input, directly learns localized temporal patterns along the time axis and achieves substantial performance improvements for certain equipment. For Id.02, the Conv1D-based model significantly outperformed the GMM and statistical feature-based Autoencoder models, achieving high detection performance across multiple evaluation metrics (Table 12). The t-SNE and KDE analyses further demonstrate clearer separation between normal and abnormal samples for this equipment (Figure 16f), indicating that the fault characteristics are more prominently expressed as localized temporal variations rather than changes in global statistical features. Similarly, for Id.06, the Conv1D-based model maintained high detection performance with stable separation between normal and abnormal data (Figure 16h).

For Id.04, although the Conv1D-based Ensemble Autoencoder improved detection performance, the gains were relatively limited compared to other equipment. The t-SNE visualization and reconstruction error distributions (KDE) show partial overlap between normal and abnormal samples (Figure 16g), suggesting that the anomalies may represent gradual degradation rather than discrete deviations from the normal state. Under such distributional characteristics, both density-based models (e.g., GMM) and reconstruction error-based unsupervised models face difficulty in defining a clear decision boundary using a single threshold. These results indicate that a single anomaly detection model cannot universally guarantee clear separation across all fault types. Therefore, both the selection of detection algorithms and the design of input representations should be adapted according to the specific fault mechanisms and signal characteristics.

Table 13 presents a comparative analysis of the computational performance of the GMM, Ensemble Autoencoder, and Conv1D–Ensemble Autoencoder models. The reported metrics—including training time, inference time, memory usage, and FLOPs—were obtained by averaging the results of five repeated runs for each model under the same hardware environment, thereby minimizing variance due to single-run fluctuations. Among the evaluated models, the GMM consistently recorded the lowest training and inference times across all equipment. The number of parameters (approximately 2–3 k) and FLOPs (approximately 3–6 k) were also minimal, indicating superior computational efficiency. Notably, the inference time ranged from 0.002 to 0.003 s, demonstrating clear suitability for real-time anomaly detection. Memory usage remained low at approximately 6–12 MB, further confirming the model’s stable operation even in resource-constrained computing environments.

The Ensemble Autoencoder exhibited a substantially higher parameter count (approximately 22 k) and greater computational complexity than the GMM; however, the training time remained relatively moderate at approximately 13–28 s. The inference time was comparable to that of the GMM (approximately 0.002–0.004 s), suggesting that reconstruction-error-based unsupervised models can maintain reasonable computational efficiency when statistical features are used as inputs. Nevertheless, memory consumption increased to approximately 430–480 MB.

In contrast, the Conv1D–Ensemble Autoencoder required the highest computational cost. The training time increased significantly to approximately 840–1300 s depending on the equipment, and the inference time ranged from 0.16 to 0.39 s. The substantial increase in parameter count (approximately 68 k) and FLOPs (approximately 11,426 k) can be attributed to the Conv1D architecture, which directly learns time–frequency patterns through convolutional operations. Memory usage also exceeded 2 GB, indicating potential constraints for deployment in edge computing environments.

In summary, the Conv1D–Ensemble Autoencoder demonstrates clear advantages when normal and abnormal data exhibit substantial overlap in their distribution or when fault characteristics manifest primarily as localized temporal patterns. In such cases, deep learning-based representation learning effectively captures discriminative features that may not be distinguishable through distribution-based analysis alone. Conversely, when anomalous data probabilistically deviates from the normal distribution in a more separable manner, the GMM maintains competitive detection performance despite its relatively simple model structure, while requiring significantly lower computational cost and memory consumption. These characteristics provide a considerable advantage in edge computing-based operational environments with constrained computational resources.

The results indicate that no single anomaly detection algorithm consistently delivers optimal performance across all equipment types and operational conditions. Consequently, the selection of an anomaly detection algorithm should be guided by a joint consideration of the distributional characteristics of normal and fault data, as well as operational constraints such as real-time requirements and available computational resources. Therefore, the primary contribution of this study is not to establish the absolute superiority of a specific algorithm but to highlight the need for an algorithm selection strategy grounded in data characteristics and deployment constraints.

## 6. Conclusions

This study proposed a lightweight unsupervised acoustic anomaly detection framework for submarine-like operational environments characterized by data scarcity, high noise levels, and constrained computing resources. Due to limitations in acquiring and sharing fault data from actual submarine onboard equipment, the publicly available MIMII dataset was adopted as a proxy platform for rotating machinery acoustics. To emulate submarine operational conditions, environmental noise components were categorized based on their physical origins and modeled as background noise, structure-borne noise with structural resonance, band-limited auxiliary noise, tonal components, and sensor noise. These components were combined and scaled to satisfy predefined SNR conditions, constructing a physics-motivated noise-augmented proxy dataset for evaluating anomaly detection algorithms under realistic noise environments.

The proposed framework quantitatively examined how input feature representation and algorithmic complexity influence anomaly detection performance and real-time deployability. In the statistical feature-based approach, MFCC features were compressed through statistical pooling to form lightweight representations and applied to both a Gaussian Mixture Model (GMM) and an Ensemble Autoencoder. To capture localized time–frequency characteristics, a data-windowing strategy using log Mel-spectrogram segments was introduced and processed using a Conv1D-based Ensemble Autoencoder. Model performance was evaluated using detection metrics (AUC, F1-score, precision, and recall) as well as computational metrics including training time, inference time, and memory usage, enabling analysis of the trade-off between detection accuracy and computational efficiency from an edge computing perspective.

Experimental results indicate that SNR-randomized noise augmentation improves model robustness under varying noise conditions. When abnormal data exhibited clear probabilistic deviations from the normal distribution (Id.00 and Id.06), the GMM achieved high detection performance with low computational cost, demonstrating strong suitability for real-time monitoring in resource-constrained environments. In contrast, when fault signatures appeared as localized temporal variations (Id.02), the Conv1D-based approach provided superior detection performance by directly modeling time–frequency patterns, albeit with higher computational requirements. For certain equipment (Id.04), overlapping reconstruction error distributions limited the effectiveness of single-threshold decision strategies, indicating that anomaly detection performance depends on signal characteristics.

Overall, the proposed framework integrates proxy data construction under security constraints, physics-motivated noise modeling, and algorithm selection considering edge-computing limitations into a unified evaluation procedure. Rather than emphasizing the superiority of a single algorithm, the study highlights the importance of jointly considering data representation, signal characteristics, and computational resources when designing acoustic anomaly detection systems. It should be noted that validation was conducted using a proxy dataset rather than real submarine measurements due to operational and security constraints. Future work will include validation of the proposed procedure using acoustic signals measured inside actual submarines to assess the reliability of the model. In particular, the noise robustness of the proposed algorithm will be evaluated under varying SNR conditions. In addition, further studies will investigate the physical validity of the noise generation assumptions by performing modal analysis to verify structural resonance parameters, such as natural frequencies and harmonic components used in the noise modeling process. Furthermore, the proposed algorithm will be integrated into an embedded system for real-time anomaly detection of rotating machinery and applied to condition monitoring of submarine seawater pumps.

## Figures and Tables

**Table 1:** Summary of representative studies including techniques, application systems, advantages, and limitations.

Categories	References	Technique	Application	Advantages	Limitations
Environmental noise modeling	Urick [7], Ross [8], Song [20], Wenz [31], Inman [32]	Underwater acoustic noise modeling	Marine acoustic environments	Provide theoretical understanding of environmental noise generation	Not designed for machinery monitoring or anomaly detection datasets
Data-driven PHM approaches	Zhao [10], Lei [11], Shao [17], Cheng [18], Yan [16], Zhang [22], Lee [23], Oliver [30]	ML/DL/transfer learning/domain adaptation/unsupervised method	Machinery fault diagnosis and monitoring	High detection capability and adaptability	Often require labeled or target-domain data; high complexity
Condition-based monitoring (physics-informed)	Niola [24]	Statistical features + Neural Network (FNN)	Hybrid electric propulsion system	Captures operating-condition behavior with low cost	Limited robustness to environmental noise
Statistical modeling approaches	Reynolds [29]	Gaussian Mixture Model (GMM)	Statistical signal modeling	Efficient and lightweight	Limited representation of complex patterns
Edge/ lightweight PHM systems	Kilinç [9] Ma [19] Yoo [27] Scudo [28]	Lightweight AI frameworks	Embedded monitoring systems	Real-time monitoring	Do not explicitly consider high-noise submarine acoustic environments

**Table 2:** Composition of normal and abnormal segments in the MIMII pump subset (Model IDs 00, 02, 04, and 06).

Machine	Model Id.	Segment for Normal Data	Segment for Abnormal Data	Sum
Pump	00	1006	143	1149
	02	1005	111	1116
	04	702	100	802
	06	1036	102	1138

**Table 3:** Parameters used in background noise generation.

Parameters	Sampling/Dist.	Value/Range	Description
α	Uniform Distribution	1.0≤α≤2.0	Spectral index of colored noise in $1/f^{\alpha }$ form
f	FFT frequency grid	$0<f<f_{s}/2$	Frequency variable
γ	Fixed predefined values	0 dB	Target signal-to-noise ratio

**Table 4:** Parameters used in structure-borne noise generation.

Parameters	Sampling/Dist.	Value/Range	Description
$f_{0,k}$	Fixed predefined values	{80, 180, 420} Hz	Representative structural resonance frequencies
$bw_{k}$		10 Hz	Standard deviation of Gaussian resonance peak
K		3	Number of resonance components
$g_{k}$	Determined from gain level	{3.0, 4.0, 6.0} dB	Resonance amplification coefficient
${\tilde{n}}_{struct}(t)$	Standard normal distribution	N(0,1)	Input excitation noise for structural shaping filter

**Table 5:** Parameters used in auxiliary noise generation.

Parameters	Sampling/Dist.	Value/Range	Description
$f_{low}$	Fixed predefined values	20 Hz	Lower cutoff frequency of band-limited filter
$f_{high}$		1500 Hz	Upper cutoff frequency of band-limited filter
${\tilde{n}}_{aux}$	Gaussian distribution	N(0,1)	Input Gaussian noise
$h_{aux}$	Band-limited filter	defined by $f_{low},f_{high}$	Spectral shaping filter

**Table 6:** Parameters used in tonal component generation.

Parameters	Sampling/Dist.	Value/Range	Description
*RPM*	Assumed	900–1800 RPM	Rotational speed range
$f_{0}$	Derived	$f_{0}=RPM/60$	Fundamental tonal frequency
K	Fixed predefined value	5	Maximum harmonic order
$A_{k}$	Harmonic decay	$A_{k}=A_{1}/k^{\beta }$	Harmonic amplitude decay
$\phi_{k}$	Uniform Distribution	$0\leq \phi_{k}<2\pi$	Random initial phase

**Table 7:** Parameters used in sensor noise generation.

Parameters	Sampling/Dist.	Value/Range	Description
${\tilde{n}}_{sensor}(t)$	Gaussian distribution	N(0,1)	Standard normal white noise
$\sigma_{s}$	Fixed predefined value	0.1	Scaling coefficient of sensor noise
$n_{sensor}(t)$	Derived	$n_{sensor}(t)=\sigma_{s}{\tilde{n}}_{sensor}(t)$	Sensor noise sequence

**Table 8:** Hyperparameters of the GMM Algorithm.

Model	Hyperparameters	Search Space	Description
Gaussian Mixture Model (GMM)	n_components	{2–30}	Number of Gaussian mixture components
	covariance_type	{‘full’, ‘tied’, ‘diag’}	Type of covariance matrix used in each Gaussian component
	reg_covar	{1 × 10^−6^, 1 × 10^−4^, 1 × 10^−3^}	Covariance regularization term added to the diagonal of covariance matrices to ensure numerical stability

**Table 9:** Hyperparameters of the Ensemble Autoencoder model.

Model	Hyperparameters	Optimization	Description
Number of experts		3	Number of Autoencoders in the ensemble
Auto-Encoder	latent_dim	{16, 32, 64}	Dimension of latent representation (compression level)
	Input dimension	42	Dimension of the MFCC statistical feature vector
	hidden_units	42 → 64 (Encoder) 64 → 42 (Decoder)	Number of neurons in each hidden layer
	learning_rate	1 × 10^−3^	Step size for weight updates
	batch_size	32	Number of samples per training iteration
	epochs	50	Total training iterations over the dataset
	Loss Function	MSE	Reconstruction loss
	Threshold	Quantile (0.95) of training errors	Decision boundary for anomaly detection

**Table 10:** Hyperparameters of the Conv1D-based Ensemble Autoencoder model.

Model	Hyperparameters	Optimization	Description
Number of experts		3	Number of Autoencoders in the ensemble
Conv1D-based Ensemble Auto Encoder	Input dimension	128 × 32	Log-mel input slice (128 mel bins, 32 time frames)
	Sliding window length	32	Fixed slice length
	Expert latent channels	{32, 64, 128}	Bottleneck channel size for each expert
	hidden_channels	128 → 64 → 32 → latent (Encoder) latent → 32 → 64 → 128 (Decoder)	Channel transition across layers
	temporal_size	32 → 16 → 8 → 8 (Encoder) 8 → 16 → 32 (Decoder)	Time dimension change via pooling and upsampling
	learning_rate	1 × 10^−3^	Step size for weight updates
	batch_size	32	Number of slices per training iteration
	epochs	50	Total training iterations over the dataset
	Loss Function	MSE	Reconstruction loss
	Threshold	Quantile (0.95) of training errors	Decision boundary for anomaly detection

**Table 11:** Specification of edge computing hardware.

Dimension	120 × 130 × 58 mm
OS	Windows 11 Home
CPU	i7-13700H
Storage	M.2-1 TB SSD
RAM	DDR4-32 G

**Table 12:** Performance comparison of unsupervised anomaly detection models under a noise-augmented dataset.

Equip. ID.	Model	AUC	Accuracy	Precision	Recall	Specificity	F1-Score
Id.00	GMM (Clean)	0.85 ± 0.002	0.90 ± 0.006	0.58 ± 0.024	0.71 ± 0.014	0.93 ± 0.008	0.64 ± 0.012
	GMM (Noisy)	0.92 ± 0.004	0.89 ± 0.006	0.75 ± 0.031	0.88 ± 0.004	0.88 ± 0.014	0.81 ± 0.011
	Autoencoder	0.98 ± 0.004	0.98 ± 0.002	0.97 ± 0.016	0.88 ± 0.014	0.99 ± 0.002	0.93 ± 0.009
	Conv1D + A.E.	0.96 ± 0.006	0.86 ± 0.026	0.89 ± 0.015	0.98 ± 0.007	0.82 ± 0.048	0.93 ± 0.009
Id.02	GMM (Clean)	0.70 ± 0.012	0.89 ± 0.005	0.39 ± 0.019	0.45 ± 0.012	0.92 ± 0.007	0.42 ± 0.011
	GMM (Noisy)	0.75 ± 0.006	0.87 ± 0.006	0.47 ± 0.024	0.47 ± 0.007	0.94 ± 0.006	0.47 ± 0.011
	Autoencoder	0.88 ± 0.013	0.94 ± 0.004	0.83 ± 0.018	0.51 ± 0.039	0.98 ± 0.001	0.63 ± 0.035
	Conv1D + A.E.	0.94 ± 0.016	0.96 ± 0.010	0.91 ± 0.012	0.94 ± 0.005	0.98 ± 0.022	0.92 ± 0.006
Id.04	GMM (Clean)	0.60 ± 0.012	0.82 ± 0.009	0.22 ± 0.022	0.18 ± 0.030	0.91 ± 0.014	0.20 ± 0.024
	GMM (Noisy)	0.64 ± 0.004	0.84 ± 0.007	0.32 ± 0.020	0.26 ± 0.019	0.92 ± 0.009	0.28 ± 0.011
	Autoencoder	0.85 ± 0.014	0.91 ± 0.006	0.77 ± 0.033	0.41 ± 0.049	0.98 ± 0.003	0.53 ± 0.045
	Conv1D + A.E.	0.94 ± 0.014	0.95 ± 0.014	0.83 ± 0.020	0.87 ± 0.015	0.84 ± 0.022	0.85 ± 0.012
Id.06	GMM (Clean)	0.88 ± 0.015	0.86 ± 0.001	0.37 ± 0.023	0.70 ± 0.026	0.88 ± 0.009	0.48 ± 0.025
	GMM (Noisy)	0.97 ± 0.001	0.93 ± 0.004	0.87 ± 0.016	0.83 ± 0.005	0.94 ± 0.004	0.85 ± 0.015
	Autoencoder	0.99 ± 0.002	0.99 ± 0.001	0.93 ± 0.001	0.99 ± 0.001	0.99 ± 0.001	0.96 ± 0.004
	Conv1D + A.E.	0.98 ± 0.001	0.89 ± 0.035	0.91 ± 0.041	0.98 ± 0.005	0.88 ± 0.057	0.94 ± 0.023

**Table 13:** Comparison of Computational Cost and Resource Usage of Unsupervised Anomaly Detection Models.

Equipment	Model	Train (s)	Inference (s)	Parameters	Flops (k)	Memory (MB)
Id.00	GMM	0.69	0.003	3059	3.1	11.87
	Ensemble AE.	12.89	0.003	22,572	44	471.6
	Conv1D–Ensemble AE.	1228.5	0.391	68,384	11,426	2185.4
Id.02	GMM	0.56	0.002	1932	6.1	6.01
	Ensemble AE.	27.76	0.003	22,572	44	438.5
	Conv1D–Ensemble AE.	1242.2	0.327	68,384	11,426	2223.2
Id.04	GMM	0.32	0.002	2576	5.6	5.56
	Ensemble AE.	20.6	0.004	22,572	44	455.8
	Conv1D–Ensemble AE.	837.7	0.167	68,384	11,426	2061.6
Id.06	GMM	0.63	0.003	2415	6.5	6.05
	Ensemble AE.	13.49	0.002	22,572	44	481.4
	Conv1D–Ensemble AE.	1300.2	0.367	68,384	11,426	2298.0

## Data Availability

The MIMII dataset used in this study can be accessed through the DOI provided in reference [27]: https://arxiv.org/abs/1909.09347 (accessed on 22 April 2026). The environmental noise datasets presented in this article are not publicly available. Although they were not directly measured in an actual submarine environment, they were constructed based on onboard equipment specifications and operational characteristics. Due to security and institutional restrictions associated with equipment-related information, public disclosure of these datasets is limited. Requests for further information may be directed to the corresponding author (jh_lee@inha.ac.kr).

## References

[1] Kim Y. (2024). Survey Research on the Utilization Level of Sensor Data for Promoting the Korean Weapon System CBM+. J. Converg. Cult. Technol..

[2] Ahmad R., Kamaruddin S. (2012). An overview of time-based and condition-based maintenance in industrial applications. Comput. Ind. Eng..

[3] Teixeira H.N., Lopes I., Braga C. Condition-based maintenance implementation: A literature review. Proceedings of the 30th International Conference on Flexible Automation and Intelligent Manufacturing.

[4] Wang H., Wang X., Tang A. (2025). Review of Deep Learning in Rotating Machinery Fault Diagnosis and Its Prospects for Port Applications. Appl. Sci..

[5] Ali A., Abdelhadi A. (2022). Condition-Based Monitoring and Maintenance: State of the Art Review. Appl. Sci..

[6] Jardine A.K.S., Lin D., Banjevic D. (2006). A review on machinery diagnostics and prognostics implementing condition-based maintenance. Mech. Syst. Signal Process..

[7] Urick R.J. (1983). The noise background of the sea: Ambient noise level. Principles of Underwater Sound.

[8] Ross D. (1976). Sound waves in liquids. Mechanics of Underwater Noise.

[9] Kilinç A., Cebesoy E.Y.Ç., Uslan V. A Short Survey on Embedded AI with Edge Computing for Maritime Applications. Proceedings of the 7th International Congress on Human-Computer Interaction, Optimization and Robotic Applications (ICHORA).

[10] Zhao R., Yan R., Chen Z., Mao K., Wang P., Gao R.X. (2019). Deep learning and its applications in machine health monitoring: A survey. Mech. Syst. Signal Process..

[11] Lei Y., Yang B., Jiang X., Jia F., Li N., Nandi A.K. (2020). Applications of machine learning to machine fault diagnosis: A review and roadmap. Mech. Syst. Signal Process..

[12] Muhammad A., Fredrik S., Pär M., Martin G., Kim B. (2022). Failure mode classification for condition-based maintenance in a bearing ring grinding machine. Int. J. Adv. Manuf. Technol..

[13] Gupta G., Ghasemian H., Janvekar A.A. (2021). A novel failure mode effect and criticality analysis (FMECA) of an industrial centrifugal pump. Eng. Fail. Anal..

[14] Mathew H., Habyarimana A., Adebiyi A. (2025). Selection and Placement of Sensors for Electric Motors: A Review and Preliminary Investigation. Energies.

[15] Nandi S., Toliyat H.A., Li X. (2005). Condition Monitoring and Fault Diagnosis of Electrical Motors—A Review. IEEE Trans. Energy Convers..

[16] Yan R., Li W., Lu S., Xia M., Chen Z., Zhou Z., Li Y., Lu J. (2024). Transfer Learning for Prognostics and Health Management: Advances, Challenges, and Opportunities. J. Dyn. Monit. Diagn..

[17] Shao H., Jiang H., Zhang H., Liang T. (2018). Electric locomotive bearing fault diagnosis using a novel convolutional deep belief network. IEEE Trans. Ind. Electron..

[18] Cheng X., Dou S., Du Y., Wang Z. (2024). Gearbox fault diagnosis method based on lightweight channel attention mechanism and transfer learning. Sci. Rep..

[19] Ma S., Sun H., Gao S., Zhou G. (2023). A real-time mechanical fault diagnosis approach based on lightweight architecture search considering industrial edge deployments. Eng. Appl. Artif. Intell..

[20] Song G., Guo X., Zhang Q., Li J., Ma L. (2023). Underwater Noise Modeling and Its Application in Noise Classification with Small-Sized Samples. Electronics.

[21] Randall R.B. (2011). Vibration Signals from Rotating and Reciprocating Machines. Vibration-Based Condition Monitoring: Industrial, Aerospace and Automotive Applications.

[22] Zhang W., Peng G., Li C., Chen Y., Zhang Z. (2017). A new deep learning model for fault diagnosis with raw vibration signals. Comput. Ind..

[23] Lee J.G., Lee J.H., Kim K.S., Kim S. (2024). Acoustic Signal-Based Fault Detection Using Variational Autoencoder and Domain Adaptation in Both Rotating and Non-rotating Machines. Asia-Pac. J. Converg. Res. Interchange.

[24] Niola V., Cosenza C., Fornaro E., Malfi P., Melluso F., Nicolella A., Savino S., Spirto M. (2025). Torque/Speed Equilibrium Point Monitoring of an Aircraft Hybrid Electric Propulsion System Through Accelerometric Signal Processing. Appl. Sci..

[25] Purohit H., Tanabe R., Ichige K., Endo T., Nikaido Y., Suefusa K., Kawaguchi Y. MIMII Dataset: Sound dataset for malfunctioning industrial machine investigation and inspection. Proceedings of the Detection and Classification of Acoustic Scenes and Events 2019.

[26] Kay S.M. (1993). Fundamentals of Statistical Signal Processing: Estimation Theory.

[27] Yoo Y., Jo H., Ban S. (2023). Lite and Efficient Deep Learning Model for Bearing Fault Diagnosis Using the CWRU Dataset. Sensors.

[28] Scudo L., Ritacco F., Caroprese E., Manco G. (2023). Audio-based anomaly detection on edge devices via self-supervision and spectral analysis. J. Intell. Inf. Syst..

[29] Reynolds D.A., Quatieri T.F., Dunn R.B. (2000). Speaker verification using adapted Gaussian mixture models. Digit. Signal Process..

[30] Oliver M., Sohn J.W. (2025). Anomaly Detection on Laminated Composite Plate Using Self-Attention Autoencoder and Gaussian Mixture Model. Mathematics.

[31] Wenz G.M. (1962). Acoustic ambient noise in the ocean: Spectra and sources. J. Acoust. Soc. Am..

[32] Inman D.J. (2014). Vibration testing and experimental modal analysis. Engineering Vibration.

[33] Harris C.M. (2002). Experimental Modal Analysis. Shock and Vibration Handbook.

[34] Carey W.M., Evans R.B. (2011). Numerical Modeling of Ambient Noise. Ocean Ambient Noise: Measurement and Theory.

[35] Lv C., Shen B., Guo X., Zhu C. Communication Design for Underwater Acoustic Positioning Networks. Proceedings of the 2019 IEEE 4th International Conference on Signal and Image Processing (ICSIP).

[36] Burdic W.S. (1991). Noise and Reverberation. Underwater Acoustic System Analysis.

[37] Julius S., Allan B., Piersol G. (2010). Digital Data Analysis Procedure. Random Data: Analysis and Measurement Procedures.

[38] Bentley J.P. (1994). Mechanical Noise and Vibration: Analysis, Measurement, and Control.

[39] Papoulis A., Pillai S.U. (2002). Stochastic Process. Probability, Random Variables and Stochastic Processes.

[40] Bently D.E., Hatch C.T. (2002). Fundamental of Vibration. Fundamentals of Rotating Machinery Diagnostics.

[41] (1995). Mechanical Vibration—Evaluation of Machine Vibration by Measurements on Non-Rotating Parts: Parts 1: General Guideline.

[42] Sturmel N., Daudet L. Signal reconstruction from STFT magnitude: A state of the art. Proceedings of the International Conference on Digital Audio Effects (DAFx).

[43] Muda L., Begam M., Elamvazuthi I. (2010). Voice recognition algorithms using mel frequency cepstral coefficient (MFCC) and dynamic time warping (DTW) techniques. J. Comput..

[44] Scrucca L. (2023). Entropy-Based Anomaly Detection for Gaussian Mixture Modeling. Algorithms.

[45] Yu B., Zhang Y., Xie W., Zuo W., Zhao Y., Wei Y. (2023). A Network Traffic Anomaly Detection Method Based on Gaussian Mixture Model. Electronics.

[46] Basha S.S., Dubey S.R., Pulabaigari V., Mukherjee S. (2020). Impact of fully connected layers on performance of convolutional neural networks for image classification. Neurocomputing.

[47] Zhou C., Paffenroth R.C. Anomaly detection with robust deep autoencoders. Proceedings of the 23rd ACM SIGKDD International Conference on Knowledge Discovery and Data Mining.

[48] Nawaz A., Shehroz S.K., Ahmad A. (2024). Ensemble of Autoencoders for Anomaly Detection in Biomedical Data: A Narrative Review. IEEE Access.

[49] Chen S., Guo W. (2023). Auto-Encoders in Deep Learning—A Review with New Perspectives. Mathematics.

[50] Kiranyaz S., Ince T., Gabbouj M. (2015). Real-time patient-specific ECG classification by 1-D convolutional neural networks. IEEE Trans. Biomed. Eng..

